# Does Domestication Cause Changes in Growth Reaction Norms? A Study of Farmed, Wild and Hybrid Atlantic Salmon Families Exposed to Environmental Stress

**DOI:** 10.1371/journal.pone.0054469

**Published:** 2013-01-31

**Authors:** Monica Favnebøe Solberg, Øystein Skaala, Frank Nilsen, Kevin Alan Glover

**Affiliations:** 1 Section of Population Genetics and Ecology, Institute of Marine Research, Bergen, Norway; 2 Department of Biology, University of Bergen, Bergen, Norway; Swansea University, United Kingdom

## Abstract

One of the most important traits linked with the successful domestication of animals is reducing their sensitivity to environmental stressors in the human controlled environment. In order to examine whether domestication selection in Atlantic salmon *Salmo salar* L., over approximately ten generations, has inadvertently selected for reduced responsiveness to stress, we compared the growth reaction norms of 29 wild, hybrid and domesticated families reared together under standard hatchery conditions (control) and in the presence of a stressor (reduced water level twice daily). The experiment was conducted for a 14 week period. Farmed salmon outgrew wild salmon 1∶2.93 in the control tanks, and no overlap in mean weight was displayed between families representing the three groups. Thus, the elevation of the reaction norms differed among the groups. Overall, growth was approximately 25% lower in the stressed tanksl; however, farmed salmon outgrew wild salmon 1∶3.42 under these conditions. That farmed salmon maintained a relatively higher growth rate than the wild salmon in the stressed tanks demonstrates a lower responsiveness to stress in the farmed salmon. Thus, flatter reaction norm slopes were displayed in the farmed salmon, demonstrating reduced plasticity for this trait under these specific experimental conditions. For all growth measurements, hybrid salmon displayed intermediate values. Wild salmon displayed higher heritability estimates for body weight than the hybrid and farmed salmon in both environments. This suggests reduced genetic variation for body weight in the farmed contra wild salmon studied here. While these results may be linked to the specific families and stocks investigated, and verification in other stocks and traits is needed, these data are consistent with the theoretical predictions of domestication.

## Introduction

Domestication is defined as the process whereby animals are adapted to the captive environment [1], [2] and therefore altered from their wild phenotype [3]. Thus, domestication is an evolutionary process, which involves adaptive genetic changes over generations [2], and is driven by directional selection for desirable traits in addition to inadvertent selection [4]. During domestication new traits have not necessarily appeared nor disappeared, but their relative expression in frequency or magnitude have been altered, causing primarily quantitative rather than qualitative changes [1], [5]. Traits that have a high heritability can therefore be modulated by selection in a relatively short evolutionary time [1], even when not deliberately selected for.

Domestication-mediated changes have been documented for body size [6]–[9], body proportion [10], fat reserves [11], [12], coloration [13], brain size [12], [14], the endocrine system [9], [15]–[17], timing of sexual maturation [18], [19], reproduction [13], [20], longevity [18], survival [21]–[23], locomotor activity [11], [24], aggressiveness [9], [10], [24]–[26], predator awareness [27], [28] and fearfulness [12], [29]–[33].

Tameness and reduced sensitivity to the domestic environment is essential for the successful domestication of animals [2], [34], and has been directly selected for in species such as silver foxes *Vulpes vulpes* [13] and rats *Rattus norvegicus* [24]. Attenuated responses in endocrine stress-related parameters, for example the release of cortisol, have been documented in domesticated animals such as the Guinea pig *Cavia aperea* f. *porcellus* [9], sheep *Ovis aries* [35], silver foxes [13], [34], [36], rats [24], rainbow trout *Oncorhynchus mykiss*, Walbaum [15] and ayu *Plecoglossus altivelis* [37]. Furthermore, strains displaying high and low cortisol responses have been successfully selected for in fish such as common carp *Cyprinus carpio* L. [38], rainbow trout [16], [39] and Atlantic salmon *Salmo salar* L. [39], [40].

Elevated cortisol levels has been documented to impose negative effects upon appetite in salmonids [41]. In a study by Fevolden and colleagues [42], rainbow trout selected for high cortisol response displayed a significantly lower growth performance than rainbow trout selected for low cortisol response, when exposed to several stressors. In Atlantic salmon, selection for fast growth has been linked with endocrine regulation of appetite [43], hence alterations in the endocrine system due to environmentally induced stress could affect the growth rate. Reduced growth as a result of repeated exposure to stress has previously been demonstrated in domesticated rainbow trout and in non-domesticated Eurasian perch *Perca fluviatilis* [44].

Domestication of Atlantic salmon was initiated in Norway by Mowi A/S and Grøntvedt Brothers in 1969, followed by the establishment of the Norwegian breeding programme, AKVAFORSK, in 1971 [45]. Today, four major breeding programs, Aqua Gen (former AKVAFORSK), Salmobreed, Mowi- and Rauma strain, collectively supply ∼ 95% of the Norwegian industrial commercial production of Atlantic salmon [6]. Atlantic salmon have high fertility rates, large phenotypic variance and a moderate generation time making the potential genetic gain through selective breeding high [46]. Like other salmonids [47], Atlantic salmon display a high heritability in growth rate, *h^2^*>0.30 [19], and the genetic gain on growth rate selection has been estimated to 10–15% per generation [19], [48]. In addition to selection for increased growth rate, defined as body weight at slaughter, late maturation, fillet quality and disease resistance has been the major breeding goals [6], [19], [48]. Behavioural traits, e.g., reduced response to human rearing, have not been included in the selection programs. Nevertheless, alterations in behavioural traits, like predator awareness [26], [28], [49], [50], has been documented in domesticated salmon.

In order to examine whether directional selection over approximately ten generations has inadvertently selected for reduced responsiveness to stress, we compared the growth reaction norms of wild, hybrid and farmed Atlantic salmon families under standard hatchery and stressful environmental conditions. Our objectives were to determine the effect of environmentally induced stress on the expression of growth and examine whether the process of domestication has affected the slopes of the growth reaction norms and not only the elevation. We predicted that when exposed to environmentally induced stress, farmed salmon would display reduced responsiveness by maintaining a relatively high growth rate in comparison to their wild counterparts.

## Methods

### Overall Design

**Figure 1:**
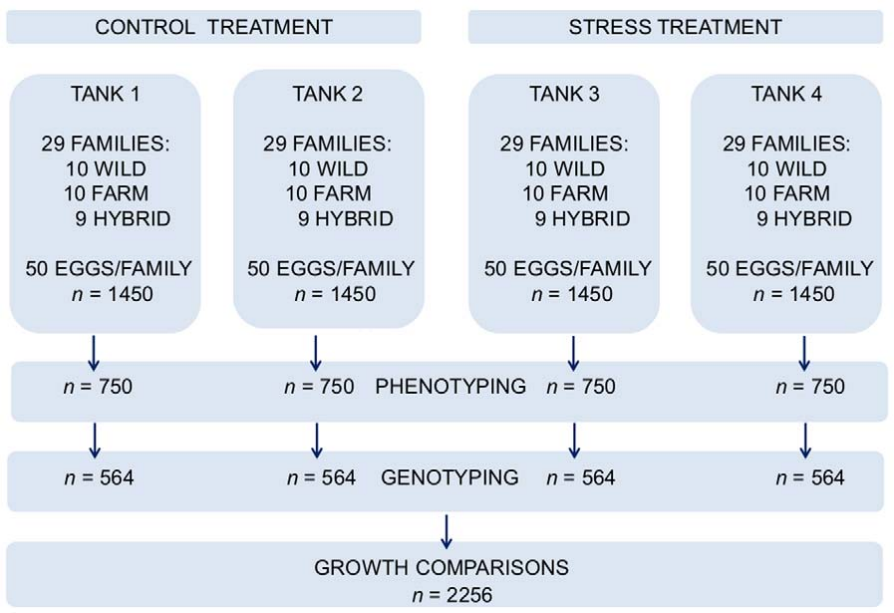
Overview of the experimental design. The experimental period lasted 14 weeks, and all sampled individuals were randomly selected. Out of the 2256 individuals genotyped, 20 individuals were excluded from the data set due to unsuccessful family assignment, growth malformations or sampling errors, leaving the total data set for growth comparisons consisting of 2236 individuals.

In order to investigate the growth reaction norms of wild, hybrid and farmed salmon in contrasting environments, families were communally reared under normal hatchery conditions and deliberately stressed tanks. In the control treatment, standard rearing conditions were provided throughout the experiment, while in the treatment group a chronic stressor was induced twice a day for fourteen weeks, until termination. Individual growth measurements were collected and all sampled individuals were assigned to family by the use of six microsatellite DNA loci. For a schematic overview of the experiment, see Figure 1.

### Experimental Crosses and Rearing

Gametes from wild Atlantic salmon originating from the Etne River (59°40′N, 5°56′E), Hordaland, and farmed salmon originating from the Norwegian Mowi strain were used to generate three cross-types for this experiment in 2009; (i) ten pure wild families; (ii) ten pure farmed families; and (iii) ten F_1_ hybrid families, generated by crossing farmed females with wild males. Thus, the hybrid families were maternal and paternal half siblings of the farmed and wild families, respectively. These three experimental groups are from now referred to as farmed (Mowi), hybrid (Mowi x Etne) and wild (Etne).

The Etne River has the largest wild salmon stock in Hordaland [51] and salmon used as parents were collected directly from the river. The Mowi strain from Marin Harvest is the oldest Norwegian farmed strain [45]. This strain was established from large multi-sea winter fish collected from the River Bolstad in the Vosso watercourse and the River Åroy, in addition to wild salmon caught in the sea outside of western Norway, near Oster fjord and Sotra [6], [52]. Phenotypic selection for growth, late maturation and fillet quality was conducted until 1999, when a family based selection program consisting of 250 females and 80 males was initiated [6]. In our study we used the offspring of the 9–10^th^ generation of selected parents.

All families were established November 17, 2009, at the hatchery located on the river Etne. Unfertilized ova and milt from 10 male and 10 female farmed salmon were collected from the Mowi breeding station located at Askøy and transported to the Etne hatchery. Wild salmon were caught by rod in October – November, 2009, transported to the hatchery located on the Etne river, and stripped upon the arrival of farmed gametes (for family crosses, see Table S1). Adipose fin clips were collected from all parental fish and scale samples from wild parents were collected and analyzed by the Norwegian gene bank for wild salmon (The Norwegian Directorate for Nature Management), to confirm that wild salmon were not escapees from farms [53].

All 30 families were incubated in the dark in single-family units, at temperatures of approximately 3.5°C (range 2.0–6.6°C), until the eyed-egg stage. Dead eggs were picked daily and February 17–18, 2010, shocked to sort out dead eggs. One hybrid family was at this point excluded from the study due to high egg mortality; hence the wild, farmed and hybrid origins were represented by 10∶10:9 families, respectively. Weight and volume measurements of eggs from all families were taken on March 17, 2010. On the same day, equal numbers of fertilized eggs per family (*n* = 50) were counted out and sorted into four replicated mixed trays (*n* = 1450; Figure 1). Experimental groups were transported to the Matre research station March 18, 2010.

The four replicates continued their incubation at the Matre hatchery at approximately 5°C (range 4–5.6°C). April 19, 2010, all four replicates were transferred to 1.5 m^3^ tanks, continuously supplied with fresh water at an average temperature of 13.2°C (range 10.7–15.6°C). All experimental groups were kept under 24 hour daily light throughout the experiment. Fry were presented with a commercial diet starting on April 22, 2010. A standard feeding table for appropriate temperatures was used to calculate the feeding ration. The fish were feed with commercial pelleted fish feed (Biomar, Myre, Norway), 12 hours per day by automatic feeders, 09.00–21.00. Pellet sizes were adjusted to the mean fish weight (W, g) after weighing a sample of 50 individuals per tank. Due to visible differences in weight among individual fish within each tank, a combination of pellet sizes were used according to supplier’s protocol to ensure that all fish were given suitable feed. Mortality was recorded daily, however dead individuals were not assigned to family.

### Ethics Statement

The experimental protocol (permit number 2648) was approved May 3, 2010, by the Norwegian Animal Research Authority (NARA). Welfare and use of experimental animals was performed in strict accordance with the Norwegian Animal Welfare Act of 19^th^ of June 2009, in forced on the 1^st^ of January 2010. All personnel involved in the experiment had undergone training approved by the Norwegian Food Safety Authority. This training is mandatory for all personnel running experiments involving animals included in the Animal Welfare Act.

### Experimental Conditions

Two tanks were reared under standard hatchery conditions (as described above) throughout the entire experiment running from June 3 - September 6–9, 2010. The two remaining tanks were subjected to a stressor, twice a day five days a week, in the same period. Stress was induced by a dramatic lowering of the water level for 30 minutes to approximately depth 3–5 cm, hence the fish density increased. Panic behaviour was observed as rapid movement within the tank. A stop watch was initiated when the water level was stabilized at the reduced level and water circulation was maintained during stressing. The water level was adjusted throughout the experimental period to control for increasing biomass (5 cm depth at termination). In all other aspects, the two treatments were given identical conditions throughout the experiment. These two treatments we hereon refer to as the control and stress treatments.

### Sampling, Genotyping and Parentage Testing

The experiment was terminated at week 14 when 750 individuals were sampled randomly from each tank over a time period of four days, one tank per day (Figure 1). All sampled individuals were euthanized with an overdose of metacain (Finquel® Vet, ScanVacc, Årnes, Norway), wet weighed, fork length measured and caudal fin clipped. Fins were preserved on 95% ethanol, and a random sample of 564 individuals from each tank was later assigned to family using DNA microsatellite markers (Figure 1).

DNA was extracted in 96 well plates using a Qiagen DNeasy®96 Blood & Tissue Kit, following procedures recommended by the manufacturer. Parental DNA was extracted twice, to ensure correct genotyping. Two randomly assigned blank wells were from this stage on included on each 96-well plate, to ensure a unique identification of the plate. Six microsatellite loci were amplified in one multiplex PCR; *SsaF43* [54], *Ssa197* [55], *SSsp3016* [GenBank# AY372820], *MHCI* [56], *MHCII* [57] and *SsOSL85* [58]. PCR products were sized-called according to the 500LIZTM standard and run on a ABI Applied Biosystems ABI 3730 Genetic Analyser. Genotypes were identified using GeneMapper V4.0., with manual control of scored alleles, and assigned to family by the use of FAP Family Assignment Program v3.6 [59]. This program has been used on several occasions for parentage testing common garden studies using these facilities [60], [61], and utilizes an exclusion-based approach to unambiguously identify parental origin. The genetic markers analysed here are routinely used in association with a genotyping service for the Norwegian legal authorities to identify the farm of origin for escapees [62], [63]. These markers have revealed very low genotyping errors in this laboratory [64]. In order to verify genotyping quality here, 70 individuals were randomly selected for re-DNA isolation and genotyping. This included individuals from all original DNA isolation plates.

### Statistical Analysis

A linear mixed effect model (LME), testing for differences in continuous response variables, were used to model variation in weight at termination between treatments and experimental groups, i.e., farmed, hybrid and wild salmon. Model selection was performed by the use of Akaike Information Criterion (AIC), calculated using restricted maximum likelihood (REML), and by the principle of parsimony the simplest model that performed best given the selected criterion was applied. The full model was fitted with treatments, experimental groups (types) and their interaction term as fixed effects and tanks, nested within treatments, as a random effect. In addition a family-related 6×6 (co)variance matrix was included to allow for heterogeneity of variance among the three experimental groups across treatments. All subsequent models were simplifications of the full model. The final model that performed best in explaining variation in weight upon termination included the fixed effects of treatment and type and their interaction term, in addition to a family-related 2×2 (co)variance matrix allowing for heterogeneity of variance cross treatments (for more information and AIC comparisons, see Table S2). The performance of wild versus farmed salmon, hybrid versus wild salmon and farmed versus hybrid salmon were compared by re-running the final model while excluding one of the three experimental groups at a time. For the re-runs, multiple comparisons were counteracted by the Bonferroni correction, giving an adjusted significance level of P<0.02.

The response variable, weight at termination, was log-transformed (log_10_). As a difference in weight between the control treatment *y* and the stress treatment *x* of value *z* would equal a greater proportion of the weight in the control treatment if the value of *y* is small than if the value of *y* is large, the log-transformations is recommended [65]–[67]. In addition, normality was achieved by the log-transformation, as the residuals of the model displayed a skewed distribution without the transformation.

P-values for the fixed effects were calculated from the F-statistics of the simplest model. The F-value and the numerator degrees of freedom (k –1, where k is the number of factor levels), were retrieved from the anova output of the LME. Denominator degrees of freedom were calculated as N – k, where N was set to the smallest sample size detected in any of the three experimental groups in any of the two treatments, i.e., 329. A significant effect of type would indicate that the farmed, hybrid and wild salmon differed in their expression of the response variable, while a significant effect of the interaction term between treatment and type would indicate that the three experimental groups differed in phenotypic plasticity in their response to treatment, i.e., their reaction norm slope [66].

In order to evaluate whether stress responsiveness was size-selective, a performance ratio (log-weight in the stress treatment *x* divided by log-weight in the control treatment *y*) was plotted against real weight in the control treatment *y*, for all families. Four pair-wise comparisons were performed per family so that both family replicates in the stress treatment were compared to the two associated family replicates in the control treatment. Under the null hypothesis there is a negative correlation between the performance ratio and *y*, hence families with large *y* values should display small values of the log-*x*/log-*y* ratio. To investigate if the experimental groups were following the null distribution by displaying negative correlations, Pearson correlations were performed between the log-*x*/log-*y* ratio and *y* for all three groups. The Pearson correlations were also used to investigate if a positive genetic correlation [68] between growth rate and stress resistance were present, as this should be detected as an overall positive correlation where each experimental group were confounded by the shape of the null distribution within the overall correlation.

In order to compare the phenotypic variance across the experimental groups, the family means of the response variable (i.e., log-weight), were compared with a median-based Levene’s test for homogeneity. Portion of phenotypic variance attributed to genetic variation were investigated by calculating heritability *h^2^* of body weight (log) as; *h^2^* =  V_A_/V_P_, where V_A_ is the additive genetic variance and V_P_ is the phenotypic variance. Variance components were estimated from the pedigree of our data by fitting a generalized linear mixed model using Markov chain Monte Carlo (MCMCglmm), i.e., the animal model [69], [70]. In the animal model, the additive genetic merit of an individual, i.e., the breeding value, is included as a random factor, *Animal* [69], [70]. Thus, V_A_ is the estimated variance in breeding values [69]. In our case, a random effect of tank was also included in the full model. Model selection was then performed by the use of the Deviance Information Criterion (DIC) and by the principle of parsimony, the tank random effect was only included if this improved the fit of the MCMCglmm (for DIC comparisons see Table S3). One model was fitted per experimental group, per treatment, i.e., six models in total.

Weakly informative priors were generated, as proposed by Wilson and colleagues [69], by equally partitioning phenotypic variance (V_P_) into the genetic and residual components, while placing little weight on the values specified by the priors, i.e., with a low degree of belief. Priors with different partitioning of the phenotypic variance between the genetic and residual components, as well as priors with stronger degree of belief, were also tested. All priors resulted in the same trend in heritability estimates among the experimental groups and treatments, and we therefore settled on the weakly informative priors yielding conservative heritability estimates.

Each model was run for 5,000,000 iterations with the first 500,000 iteration excluded as burn-in, and was thereafter sampled at every 500 iteration. Convergence of the model was checked by calculating autocorrelations among the samples of the posterior distributions [69]. As a measure of precision of the heritability estimate, credibility intervals were calculated as 95% highest posterior density (HPD) intervals.

All statistical analysis was performed using R ver. 2.15.1 (R Development Core Team; www.r-project.org) with critical *P*-values set to 0.05, unless otherwise stated. Data exploration were performed in accordance with the protocol by Zuur et al. [71]. LMEs were fitted using the *lmer* function in the lme4 package [72], and Levene’s tests were performed using the *leveneTest* function in the car package [73]. Heritability and additive genetic variance were estimated using the MCMCglmm package [74], while the HPD intervals were calculated using the *HPDinterval* function in the lme4 package [72].

## Results

### Genotyping and Parentage Testing

Among the 750 fish sampled per tank, 564 were randomly selected for parental assignment (Figure 1). Of the 70 fish randomly selected for re-genotyping, in order to verify genotyping and sample-handling accuracy, all gave identical genotype and parentage assignments on the second analysis. Of the 2256 individuals that were chosen for parentage analysis, 2243 were unambiguously indentified to family. This gave 9–29 individuals/family/tank (Table S4). All of the 13 individuals that could not be unambiguously assigned to family displayed overlapping composite genotypes between family pairs. These individuals were simply removed from the data set. After parentage assignment, seven individuals were identified as outliers and post hoc excluded from the data set, hence leaving the total data set consisting of 2236 individuals. All excluded outliers displayed growth malformations or were caused by sampling errors. Of the 2256 individuals genotyped, 70 were identified trisomic at one or more loci. Individual weight of these trisomic fish was compared to their mean family weight in their respective tanks, and classified as either above (*n* = 38) or below (*n* = 32) the family average (data not presented). These trisomic individuals were not smaller, nor larger than the diploid individuals within the same family (G-test: G = 0.51, df = 1, P = 0.47). Consequently, these individuals were included in the data set.

### Mortality and Growth in Tanks

Mortality was low in all tanks throughout the experimental period and identical between treatments (G-test: G = 0.26, df = 1, P = 0.63; Table 1).

**Table 1:** Growth measurements and mortality of *Salmo salar* L. in all tanks throughout the experimental period.

			Weight During Experiment (g)				Measurments at termination (week 14)			Mortality		
Treatment	Tank	*n*	Week 0	Week 3	Week 6	Week 11	MeanK ± SD	Mean L(cm) ± SD	Mean W(g) ± SD	∼week 0	week0–14	Total (%)
Control	1	561	1.32	2.54	4.91	16.60	1.31±0.09	11.54±2.27	22.64±12.06	81	43	8.55
	2	560	1.34	2.57	5.25	16.10	1.27±0.08	11.65±2.27	22.61±12.05	56	69	8.62
Stress	3	560	1.27	2.30	3.93	12.10	1.23±0.09	10.51±2.23	16.44±09.50	37	40	5.31
	4	555	1.26	2.43	4.59	11.90	1.24±0.10	10.56±2.34	17.06±10.20	40	65	7.24

Condition factor (K), length (cm) and weight (g), with standard deviations, and mortality (absolute and percent). Weight during experiment based upon bulk weight of 50 individuals.

**Figure 2:**
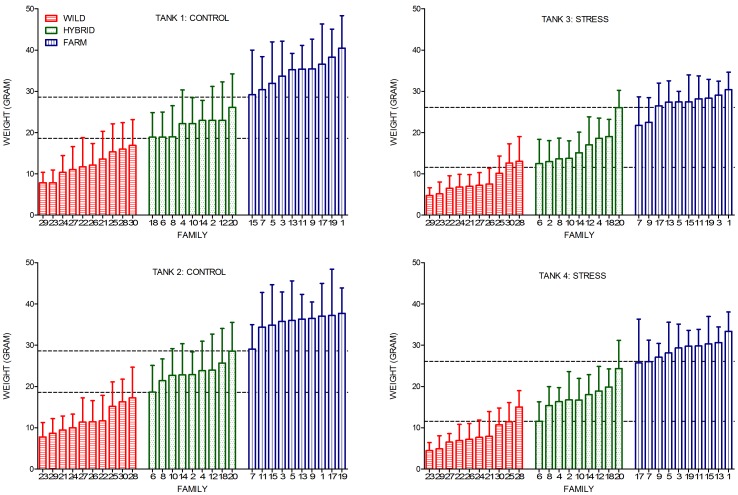
Mean family weight. Mean family weight (g) of wild, hybrid and farmed families in all four tanks. Farmed families were larger than wild families, while hybrid families were displayed at an intermediate range. All groups grew better in the control treatment, than in the stress treatment. In the control treatment there was no overlap in the weight range of families of farmed, hybrid and wild origin. Lines represent the mean of the smallest and largest hybrid family within each treatment. Error bars show the standard deviation.

Treatment specific growth was already clear in the early stages of the experiment (Table 1). Upon termination, the mean weight of individuals in the stress treatment was significantly lower than for individuals in the control treatment, with replicates displaying similar growth (LME Treatment: F_1, 327_ = 164.58, Sum Sq = 5.17, P<0.001; Table 1; Figure 2). Thus, significant tank effects were not observed, and the inclusion of the random effect of tanks nested within treatments did not improve the fit of the model.

### Group and Family Growth

**Figure 3:**
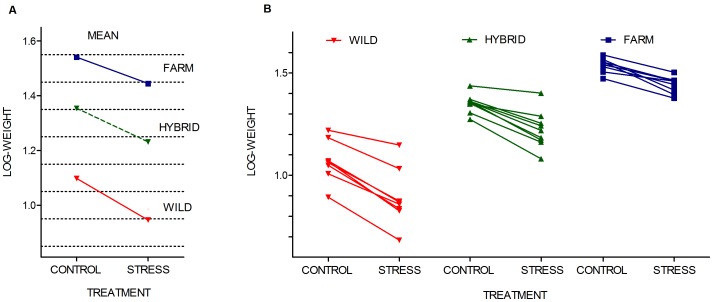
Growth reaction norms of Atlantic salmon. Log-weight norm of reaction across treatments at the a) group level and b) family level for salmon of wild, hybrid and farmed origin. Replicated tanks are pooled. The elevation of the reaction norms was significantly different in the farmed, hybrid and wild salmon. Wild salmon displayed significantly steeper slopes than the farmed salmon, while hybrid salmon displayed slopes insignificantly different to the farmed and wild salmon.

At the start of the experiment, farmed, hybrid and wild individuals were approximately 1.60, 1.38 and 1.30 grams, respectively. These data are based upon the bulk weight of 50 individuals, sampled from mixed-family single-group tanks that were reared in parallel to this experiment. Upon termination of the experiment, farmed salmon were significantly larger than wild salmon, with hybrids displaying an intermediate weight, in both treatments (LME Type: F_2, 326_ = 108.34, Sum Sq = 6.80, P<0.001; Table 2; Figure 2). Thus, the elevations of the reaction norms were significantly different in all three groups (Figure 3; Table S5).

**Table 2:** Growth measurements of sampled *Salmo salar* L. of wild, hybrid and farmed origin.

				Measurments at Termination (week 14)					Weight difference	
Group	Treatment	Tank	*n*	Mean K ± SD	Mean L (cm) ± SD	Mean W (g) ± SD	*n*	Mean W (g) ± SD	Absolute (g)	Percent %
Wild	Control	1	212	1.29±0.11	09.56±1.64	12.25±6.28	425	11.98±6.07	3.88	32.47
		2	213	1.23±0.09	09.56±1.57	11.71±5.86				
	Stress	3	209	1.19±0.10	08.47±1.46	07.99±4.45	435	08.10±4.65		
		4	226	1.19±0.12	08.50±1.59	08.20±4.82				
Hybrid	Control	1	162	1.31±0.07	11.75±1.54	22.23±7.58	339	22.85±7.47	5.66	24.77
		2	177	1.27±0.07	12.11±1.40	23.41±7.35				
	Stress	3	177	1.24±0.08	10.76±1.53	16.41±6.43	351	17.19±6.39		
		4	174	1.26±0.06	11.09±1.41	17.98±6.27				
Farm	Control	1	187	1.35±0.06	13.60±1.29	34.78±8.50	357	35.08±8.27	7.31	20.84
		2	170	1.31±0.06	13.81±1.24	35.42±8.02				
	Stress	3	174	1.27±0.07	12.68±1.14	26.60±6.00	329	27.77±6.16		
		4	155	1.30±0.06	12.98±1.14	28.94±6.15				

Condition factor (K), length (cm) and weight (g), with standard deviations, and weight difference between treatments (absolute and percent).

There was no overlap in mean family weight between families of farmed, hybrid and wild origin, in the control treatment (Figure 2). In the stress treatment there was overlap in mean weight between four wild/hybrid families, and between three hybrid/farm families (Figure 2; Table S4).

All experimental groups grew better in the control treatment than in the stress treatment, thus displaying negative reaction norm slopes (Figure 3, Table S5). Hence at the family level, individuals in the stress treatment were significantly smaller than their siblings in the control treatment. Exceptions were detected in one farmed family (family 15) where the mean family weight in one of the stress treatment replicates was higher than in one of the control treatment replicates, and in one hybrid family (family 18) where the mean family weight in one of the control treatment replicates was lower than in both stress treatment replicates (Figure 2; Table S4).

### Growth Reaction Norms

The effect of environmentally induced stress upon growth were significantly different between the farmed, hybrid or wild salmon (LME Interaction term: F_2, 326_ = 6.19, Sum Sq = 0.39, P = 0.002). Between the wild and farmed salmon, the interaction term between treatments and genetic origin were significantly different (LME Interaction term: F_1, 327_ = 13.35, Sum Sq = 0.43, P<0.001, Bonferroni P<0.02). Wild salmon displayed stronger plasticity in their response to the stress treatment as the reaction norm slope of the wild salmon was significantly steeper than the slope of the farmed salmon (Figure 3A). Thus, farmed salmon were less affected by the experimental stressful conditions than their wild counterpart. However, the interaction between treatments and genetic origin were not significantly different between the wild and hybrid salmon (LME Interaction term: F_1, 327_ = 3.82, Sum Sq = 0.14, P = 0.03, Bonferroni P<0.02;) nor between the hybrid and farmed salmon (LME Interaction term: F_1, 327_ = 1.48, Sum Sq = 0.03, P = 0.16, Bonferroni P<0.02). Thus, the slope displayed by the hybrid salmon was not significantly different from the slope displayed by neither the wild nor the farmed salmon (Figure 3A).

**Figure 4:**
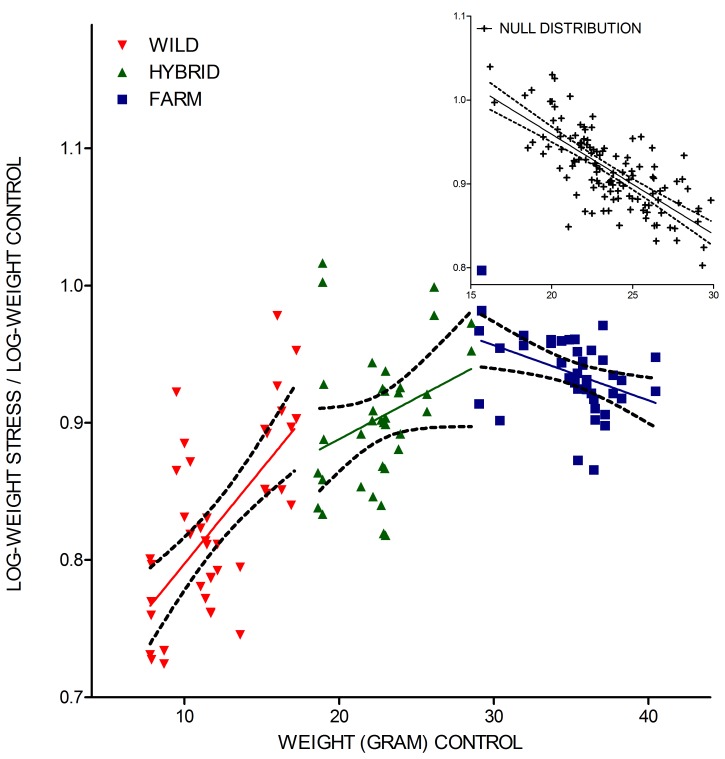
Pearson correlations between performance ratio and mean family weight in the control treatment. Performance ratio (mean log-weight in the stress treatment *x* relative to mean log-weight in the control treatment *y*) plotted against the mean family weight in the control treatment *y* for wild, hybrid and farmed families, including regression lines. A significant positive correlation, a non-significant correlation and a significant negative correlation were detected for the wild, hybrid and farmed families, respectively. The regression line is shown with a 95% confidence interval. The null hypothesis, illustrated by 120 randomly generated values of the true mean of both treatments (SD = 10% of the mean), illustrates a negative correlation under the null distribution.

As a result of the steeper reaction norm slopes, wild families displayed lower performance ratios (log-*x*/log-*y*) than the hybrid and farmed salmon, despite their low weight in the control treatment *y* (Figure 4). Farmed salmon displayed a negative correlation between the performance ratio and weight in the control treatment (*n = *40, Pearson r = −0.41, P = 0.002) hence being confounded by the null distribution (Figure 4). Wild families displayed a significant positive correlation (*n = *40, Pearson r = 0.66, P<0.001; Figure 4), while hybrid families displayed an insignificant correlation between the performance ratio and weight in the control treatment (*n = *36, Pearson r = 0.31, P = 0.07; Figure 4). Overall, these results deviate from the null hypothesis (Figure 4), and demonstrate that the displayed differences in stress responsiveness of farmed and wild salmon were not an artefact caused by differences in body weight per se, nor by a positive genetic correlation between growth rate and stress resistance [68].

### Variance and Heritability

The phenotypic variance in the family means of the response variable, i.e., the log transformed body weight, was significantly different in the three experimental groups in the control treatment (Levene’s Test: Tank 1: F = 5.01, df = 2, 26, P = 0.01; Tank 2: F = 5.87, df = 2, 26, P = 0.01; see Figure 3B). In the stress treatment variance was significantly different in the three experimental groups in one of the replicated tanks, while borderline insignificant in the other replicate (Levene’s Test: Tank 3: F = 2.91, df = 2, 26, P = 0.07; Tank 4: F = 4.38, df = 2, 26, P = 0.02; see Figure 3B).

Wild salmon displayed higher heritability *h^2^* of body weight (log), than the hybrid and farmed salmon (Table 3). Thus, a larger portion of the observed phenotypic variance was attributable to genetic variation in the wild as compared to the farmed and hybrid salmon. However, broad and overlapping credibility intervals, i.e., 95% highest posterior density intervals, were detected between the experimental groups, in both treatments. In the control treatment hybrids displayed similar heritability estimates as the farmed salmon, while in the stress treatment hybrids were displayed at an intermediate level. All experimental groups displayed higher heritability estimates in the stress treatment, than in the control treatment (Table 3).

**Table 3:** Heritability of the trait body weight in salmon of wild, hybrid and farmed origin.

			HPD interval	
Treatment	Group	*h^2^*	Lower	Upper
Control	Farm	0.14	0.05	0.44
	Hybrid	0.14	0.04	0.42
	Wild	0.29	0.05	0.80
Stress	Farm	0.18	0.07	0.57
	Hybrid	0.34	0.05	0.81
	Wild	0.60	0.15	0.89

Heritability *h^2^* of the trait body weight (log) calculated using the animal model, implemented by MCMCglmm. The upper and lower 95% highest posterior density HPD intervals represents the credibility intervals of the estimated *h^2^*. Models were fitted separately for each experimental group, in both treatments. In addition to the breeding value, *Animal*, the random effect of tank was included when this improved the fit of the MCMCglmm. Model selection was performed by the use of the Deviance Information Criterion (for DIC comparisons see Table S3).

## Discussion

This study reports the growth reaction norms of farmed, hybrid and wild Atlantic salmon, at the family level, that have been communally reared in two contrasting environments. The main results can be summarised as: (i) farmed salmon outgrew hybrid and wild salmon in both treatments, thus the elevation of the reaction norms differed among the groups; (ii) mean family weight did not display overlap between farmed, hybrid and wild families in the control environment; (iii) phenotypic plasticity in the response to treatment, the growth reaction norm slope, was significantly smaller for salmon of farmed origin than for salmon of wild origin; (iv) wild salmon displayed a higher heritability *h^2^* of the trait body weight, than the hybrid and farmed salmon; (v) heritability of body weight was greater for all experimental groups in the stressed as opposed to the control environment; (vi) hybrid salmon displayed intermediate values for all growth measurements.

Given that farmed salmon families maintained a relatively higher growth rate than the wild salmon families in the stressed tanks, it is concluded that the process of domestication has resulted in reduced responsiveness to stress in Atlantic salmon.

### Growth

Under standard hatchery conditions, farmed salmon outgrew wild salmon by 1∶2.96. Hence in ten generations, the growth rate of commercially reared salmon at the freshwater stage has increased almost three-folded compared to its wild origin. This is to our knowledge the strongest growth rate difference detected between salmon of wild and farmed origin. Previous studies have detected a difference in total body weight between wild and farmed salmon in the freshwater stage of 1∶1.3 in the 1^st^ generation of selection [75], [76], 1∶1.96 in the 5^th^ generation [8] and 1∶2.4 in the 7–8^th^ generation [6], thus indicating that genetic gain for increased growth still can be selected for. Hybrid salmon were displayed at an intermediate level, being outgrown by 1∶1.54, thus supporting previous growth studies [6], [77]. In the stress treatment growth was lower for all groups, and farmed salmon outgrew hybrid and wild salmon by 1∶1.61, and 1∶3.42, respectively.

In our study where salmon of all origins were reared communally, any potential group specific tank effects were avoided [78]. However, we cannot rule out if differences in competitive ability or aggressiveness between the strains could, in part, affect these results. Therefore this study could be complemented by similar growth comparison of these or other strains, when reared in both mixed- and single stock tanks.

### Stress

In our study, smaller reaction norm slopes and therefore less plasticity in the response to treatment were displayed in the farmed salmon and we therefore conclude that farmed salmon display reduced responsiveness to stress, in comparison to their wild counterparts. This is consistent with the theory of domestication, as decreased sensitivity to the domestic environment has been documented to be a major part of domestication, either through directional or inadvertent selection [1], [2], [34]. Human handling or rearing routines associated with the domestic environment could trigger acute stress responses in salmonids causing an increase in activity (flight or flight response). As this would increase energy expenditure, potential resources for growth would be extracted, and therefore have a negative effect upon growth rates. Poststress reduced feeding rate has also been documented in salmonids [10], [15], [26]. In our study, impaired growth rates were detected in the stressed salmon of all origin, which could have been caused by increased energy expenditure associated with the detected increase in locomotor activity, by impaired feed intake, or by a combination of both. Thus, selection for increased growth rate is likely to inadvertently have selected for reduced stress responsiveness, as stressed individuals would display impaired growth rates, and therfore would not be selected among the brood fish to propagate the next generation. Consistent with this theory, the farmed salmon in this study maintained a relative higher growth rate in the stressed environment compared to the wild salmon.

Reduced growth performance when exposed to repeated stress has been documented in both domesticated and non domesticated fish species, together with elevated cortisol levels [44]. Cortisol is well documented as a stress marker in fish [79] and in salmonids the estimated heritability is high, i.e., *h^2^*>0.50 [42], [80]. Reduced resting and poststress cortisol levels have been documented in domesticated relative to wild rainbow trout [15] and stains of high and low stress-induced cortisol levels have been successfully selected for in rainbow trout [16], [39], [81] and Atlantic salmon [39]. However, selection for low cortisol levels was never included in the Norwegian salmon breeding program.

Reduced stress responsiveness due to inadvertent selection is documented in species domesticated for increased growth, e.g., chickens *Gallus domesticus* [29], [30], [82], [83], and vice versa, e.g., rats [24], [84]. Due to a trade off between growth and mortality rates in the wild [85], absence of natural predators in the domesticated environment inadvertently selects for increased growth [85]–[87]. Traits that are energetically costly to maintain will over time become obsolete if they no longer provide a competitive edge, due to relaxed selection [5]. Hence, traits needed for survival in the wild, e.g., predator avoidance, start to decrease in frequency and/or magnitude in the domesticated predator free environment and reduced anti-predator responses in farmed salmonids is documented in several comparative studies [10], [26], [28], [50].

### Plasticity

Phenotypic plasticity is the general change in phenotype caused by changes in the environment, whereas the norm of reaction is the specific form of that change [88]. Thus, the slope of the reaction norm represents the plasticity of the investigated trait [89]. Farmed, hybrid and wild salmon all responded significantly to the stress treatment by displaying negative growth reaction norm slopes, hence displaying phenotypic plasticity in growth as a response to altering stress levels. However, the slope of the reaction norms differed among the groups, as farmed salmon, compared to the wild salmon, displayed reduced responsiveness to stress and therefore less negative slopes (i.e., flatter) (Figure 3). This documents a differing degree of plasticity in the farmed and wild salmon, with farmed salmon displaying reduced plasticity in the investigated trait under these environmental conditions. In addition, the variance of the family means differed among the three experimental groups, in both treatments, with farmed families displayed less variation in the reaction norms across treatments, than the hybrid and wild salmon (see Figure 3B).

Comparative studies of reaction norms in farmed and wild crosses have revealed differing results. In farmed and wild salmon exposed to differing temperature regimes, differing elevations, although similar slopes, were detected in the reaction norms for survival to hatch [90]. For time to 50% hatch, as well as yolk sack weight, the slopes of the reaction norms differed among the crosses, whereas for length at hatch, no differences in the reaction norms were detected [90]. Backcrossed farmed and wild salmon from the same region displayed similar reaction norm slopes for compensatory growth, only differing in elevation [91] and in a study investigating acid tolerance, changes in both the slope and the elevation of the reaction norm for survival and growth at the alvin stage were revealed, although no difference were detected at the parr stage [92].

Data from our common garden experiment display reduced phenotypic plasticity in the farmed salmon studied here and we therefore suggest that domestication might lead to a reduction in the genetic based plasticity of growth in farmed Atlantic salmon. However, phenotypic plasticity is not always adaptive, and non-adaptive plasticity in response to varying environments could influence the mean of the phenotypic trait, as well as the expression of variance [93], [94]. For instance, cryptic genetic variation that is not expressed under normal conditions could be revealed in stressful environmental conditions [93]. Thus, if the environment is perceived differently among genotypes, a non-adaptive response could result in the expression of differing levels of plasticity, depending on how divergent the stressful environment is relative to the genotypes optimal environment. As our experimental conditions resemble the farmed environment more than the wild, similar studies across different farmed-wild environmental gradients could provide more information on whether the observed reduction in plasticity will be expressed in other environments.

### Genetic Variation

Higher heritability *h^2^* of the trait body weight was detected in the wild contra the farmed and hybrid salmon, in both environments. Furthermore, heritability increased in the stress treatment in all experimental groups. The increase in genetic variation in the stress treatment could be caused by phenotypic variation among genotypes being suppressed in favourable conditions such as the control environment [95]. Hence, genetic variation could appear more clearly under unfavourable conditions such as the stressed environment. Overall, our results suggest that the farmed salmon studied here displayed reduced genetic variation for body weight, in comparison to the wild salmon. However, broad and overlapping credibility intervals of the heritability estimates were detected in the experimental groups in both treatments. This is probably due to the fact that our experimental design was not optimal to accurately estimate heritability. Thus the trend in the heritability estimates, maybe more than the isolated *h^2^* values, reflects the difference in genetic variation among the experimental groups.

Reduced genetic variation in domesticated salmon strains has been documented in neutral genetic markers [96]–[99] and indications of reduced genetic variation in susceptibility to the sea louse *Lepeophtheirus salmonis* has been suggested in farmed salmon smolts [100]. Reduced genetic variation in a quantitative trait, as detected in our study, is supported in a recent common garden study indicating a genetically based anti-predator response in Atlantic salmon, where farmed salmon displayed a lower variance in the studied trait, in comparison to wild salmon [28]. In contrast, increased genetic variation in a quantitative trait, allelic variation at the major histocompatibility complex (MHC) class II, has been documented in the domesticated Australian Atlantic salmon relative to their Canadian ancestor [101], although at the same time displaying reduced non-coding genetic variation [99]. As reduced MHC variation is likely to have negative effects upon disease resistance, variation is assumed to have been maintained in this farmed strain trough intense balancing selection during domestication [102].

The documentation of both decreased and increased genetic variation in domesticated salmon indicates the importance of interpreting genetic variation in context of the studied trait and its importance to fitness in the respective environment. In context for our study, reduced genetic variation for body weight were detected in the farmed salmon. This is consistent with the theoretical predictions of domestication, as this trait has been the primary target for the breeding programs [19] for approximately ten generations. However, if reduced genetic variation is a general feature in domesticated salmon, or if these results are specific to our study, remains to be tested in more strains and/or traits in differing environments.

### General Implications

Genetic interaction between farmed escaped salmon and wild conspecifics represents one of the major environmental challenges faced by the aquaculture industry. Although the successful genetic introgression of farmed escaped salmon has been documented in several rivers [103]–[109], detecting introgression of farmed salmon in natural populations is not without technical difficulties [110]. Thus, although a recent genome-scan revealed a panel of single nucleotide polymorphism markers (SNP) that appear collectively diagnostic on the farmed/wild interface [111], there is still a pressing need to identify more robust genetic markers to identify farmed and wild salmon in order to be able to properly evaluate the impact that escapees has had on wild populations. Thus, controlled experiments, such as the present study, will not only be invaluable in elucidating the underlying genomic differences between farmed and wild salmon, when combined with linkage mapping, they may also contribute to the identification of genetic markers associated with domestication.

## Supporting Information

Table S1 **Family design and parental origin of** ***Salmo sala*** **r L. families used in the experiment.**
(XLS)

Table S2 **AIC comparison of linear mixed effects models fitted for log-weight (g) at the time of termination.**
(XLS)

Table S3 **DIC comparisons of the animal model implemented by MCMCglmm.**
(XLS)

Table S4 **Family growth measurements of** ***Salmo salar*** **L. of wild, hybrid and farmed origin in all tanks.**
(XLS)

Table S5 **Summary of the best fit linear mixed effect models testing for differences in log-weight of wild versus farm salmon, hybrid versus wild salmon and farm versus hybrid salmon.**
(XLS)
